# Insulinoma in a Young Patient With Focal Seizures: A Case Report of a Neurological Diagnostic Challenge

**DOI:** 10.1002/ccr3.71931

**Published:** 2026-01-24

**Authors:** María Valerio López, Christopher Kaleb Romero Ríos, Mohammed Zahran, Allan Mariano Bodan Campbell, Amilcar Alfaro, Jorge Chamorro Flores

**Affiliations:** ^1^ Department of Internal Medicine Hospital Manolo Morales Peralta Managua Nicaragua; ^2^ School of Medicine Hospital Militar Escuela “Dr. Alejandro Dávila Bolaños” Managua Nicaragua; ^3^ Department of Hepatopancreatobiliary Surgery Hospital Manolo Morales Peralta Managua Nicaragua; ^4^ Department of Radiology Hospital Manolo Morales Peralta Managua Nicaragua

**Keywords:** focal seizures, hypoglycemia, insulinoma, neuroendocrine tumor, neuroglycopenia, pancreatectomy

## Abstract

Insulinomas can mimic refractory epilepsy, leading to diagnostic delays. Hypoglycemia should be ruled out in patients with fasting‐associated focal seizures. Timely diagnosis via Whipple's triad and surgical resection offers a complete cure and reversal of neuropsychiatric symptoms.

## 1 Introduction

Insulinoma is the most common functional pancreatic neuroendocrine tumor, although it is rare, with a global incidence estimated at 1–4 cases per million people per year [1]. Its pathophysiology involves autonomous insulin secretion that is not suppressed by hypoglycemia, leading to recurrent episodes, typically occurring during fasting or physical exertion [2, 3]. Since the brain depends almost exclusively on glucose as an energy source, neuroglycopenia disrupts neuronal function and produces the most severe clinical manifestations [4]. Whipple's triad remains the cornerstone of diagnosis: (1) symptoms compatible with hypoglycemia, (2) low plasma glucose during symptoms, and (3) relief of symptoms after glucose administration [5]. Neuroglycopenic symptoms including confusion, behavioral changes, amnesia, and seizures make insulinoma a “great imitator” of neurological disorders [6]. We report the case of a young patient who was treated for epilepsy for 18 months before the underlying etiology, an insulinoma, was identified. This case highlights the importance of considering metabolic causes in atypical or refractory seizures, emphasizing that timely diagnosis and treatment can lead to complete cure.

## 2 Case History/Examination

An 18‐year‐old male with no significant past medical history was referred to our unit in September 2023. He had an 18‐month history of focal motor‐onset seizures, attributed to the right frontal lobe. The episodes consistently occurred in fasting states and were preceded by a prodrome of malaise, weakness, and profuse sweating.

He had been treated with carbamazepine 200 mg every 8 h without adequate seizure control. His records documented two prior hospitalizations where severe hypoglycemia was recorded (35 and 45 mg/dL), with complete symptom remission after intravenous dextrose administration; however, no endocrine workup had been performed.

The current admission was prompted by progressive cognitive decline and an increase in seizure frequency. On examination, the patient was disoriented but otherwise had a normal physical exam. Initial laboratory tests showed preserved renal and liver function and normal electrolyte levels (Table 1).

**TABLE 1:** Baseline laboratory findings at initial clinical evaluation.

Parameter	Patient value	Reference range
Random serum glucose	78 mg/dL	70–110 mg/dL
Hemoglobin	14.5 g/dL	13.5–17.5 g/dL
Creatinine	0.9 mg/dL	0.7–1.2 mg/dL
Sodium	140 mEq/L	136–145 mEq/L
Potassium	4.1 mEq/L	3.5–5.1 mEq/L
Serum calcium	9.5 mg/dL	8.6–10.3 mg/dL
AST	25 U/L	10–40 U/L
ALT	30 U/L	7–56 U/L

*Note:* Laboratory parameters including random serum glucose, hemoglobin, renal function, electrolytes, serum calcium, and liver enzymes were within normal reference ranges at presentation.

## 3 Differential Diagnosis, Investigations, and Treatment

The differential diagnosis included primary focal epilepsy, hypoglycemia due to endogenous or exogenous causes, and other metabolic or structural disorders. The history of fasting‐associated episodes, adrenergic prodromal symptoms, and complete resolution after glucose administration strongly suggested a hypoglycemic etiology.

During hospitalization, the patient experienced another episode of altered consciousness with a glucose level of 30 mg/dL. A supervised 72‐h fasting test, the diagnostic gold standard [7] was initiated. At 48 h, he developed severe neuroglycopenic symptoms with a venous glucose of 30 mg/dL. A critical sample was obtained before intravenous glucose administration, leading to complete symptom reversal and fulfillment of Whipple's triad. The results of the critical sample confirmed endogenous hyperinsulinism (Table 2).

**TABLE 2:** Critical laboratory results obtained during the supervised fasting test.

Parameter	Patient value	Diagnostic criterion (Endocrine Society) [7]
Plasma glucose	30 mg/dL	< 55 mg/dL
Serum insulin	15.2 μU/mL	≥ 3.0 μU/mL
C‐peptide	2.5 ng/mL	≥ 0.6 ng/mL

*Note:* The table summarizes plasma glucose, serum insulin, and C‐peptide levels at the time of hypoglycemia, meeting the diagnostic criteria for endogenous hyperinsulinemic hypoglycemia.

**FIGURE 1:**
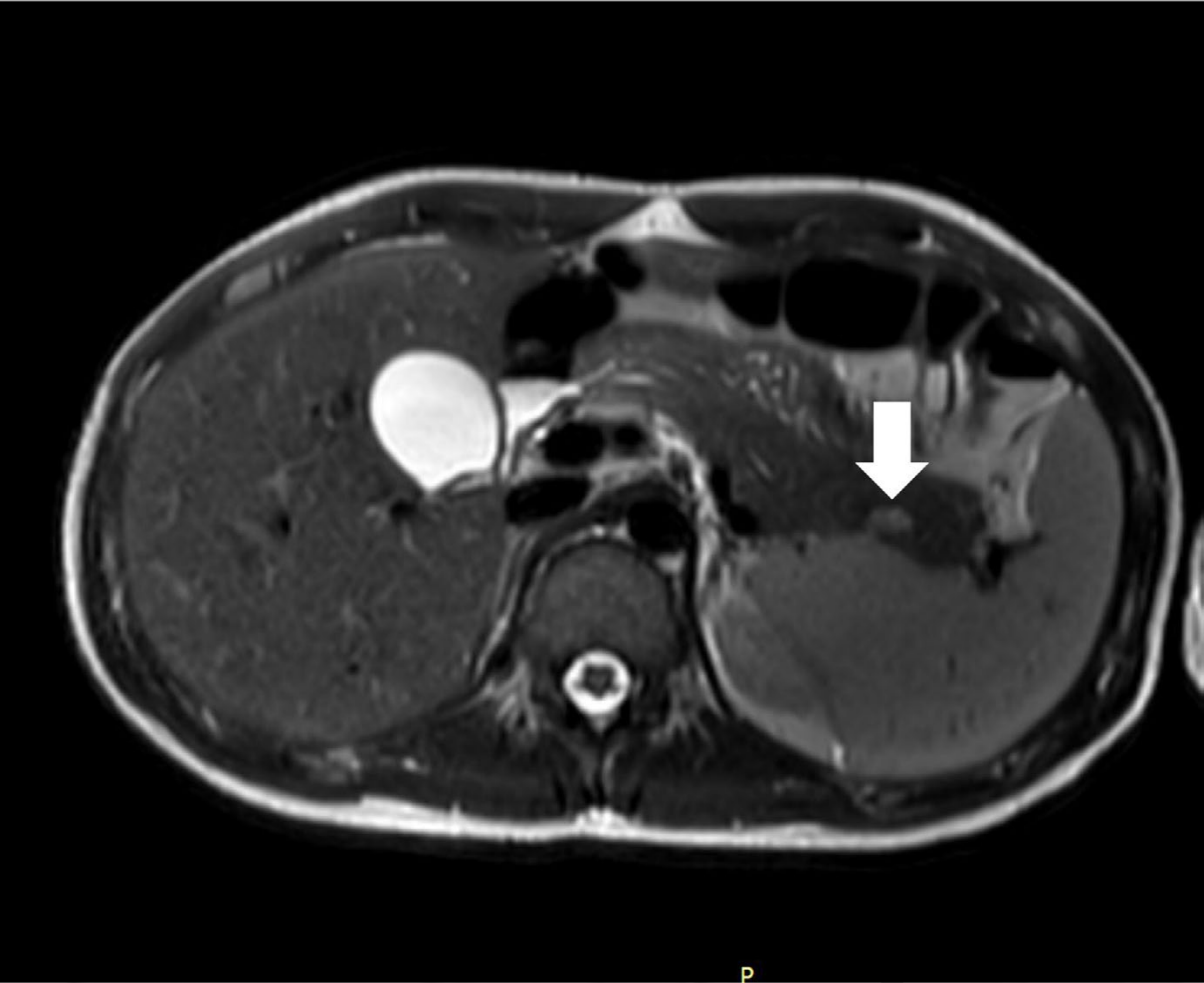
Abdominal magnetic resonance imaging. Axial T2‐SPAIR gradient‐echo MRI showing a hypervascular intrapancreatic nodule at the posteroinferior aspect of the pancreatic tail (white arrows).

After biochemical confirmation, contrast‐enhanced abdominal magnetic resonance imaging (MRI) revealed a well‐defined, hypervascular nodule in the pancreatic tail, a characteristic finding of insulinoma (Figure 1). No metastases were identified.

**FIGURE 2:**
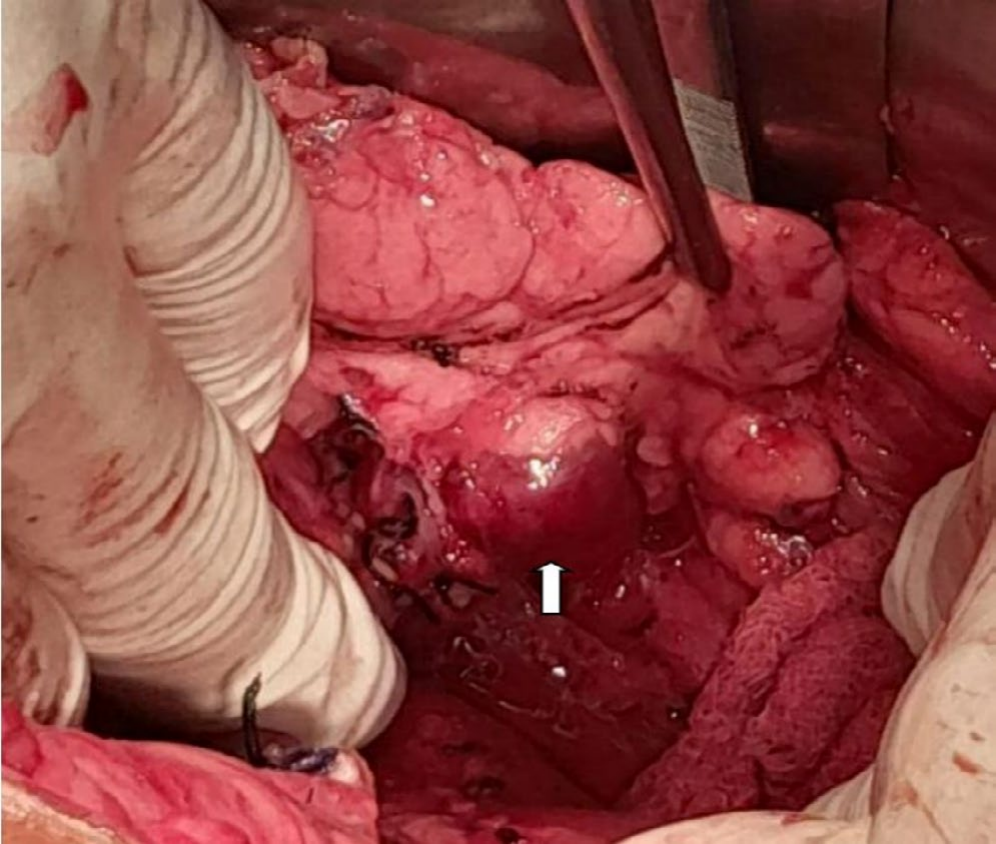
Intraoperative image. White Arrow showing a hypervascular mass at the pancreatic tail.

The patient underwent laparoscopic distal pancreatectomy (Figure 2). Postoperative recovery was uneventful, with complete resolution of hypoglycemic episodes and seizures. Carbamazepine was discontinued prior to discharge.

**FIGURE 3:**
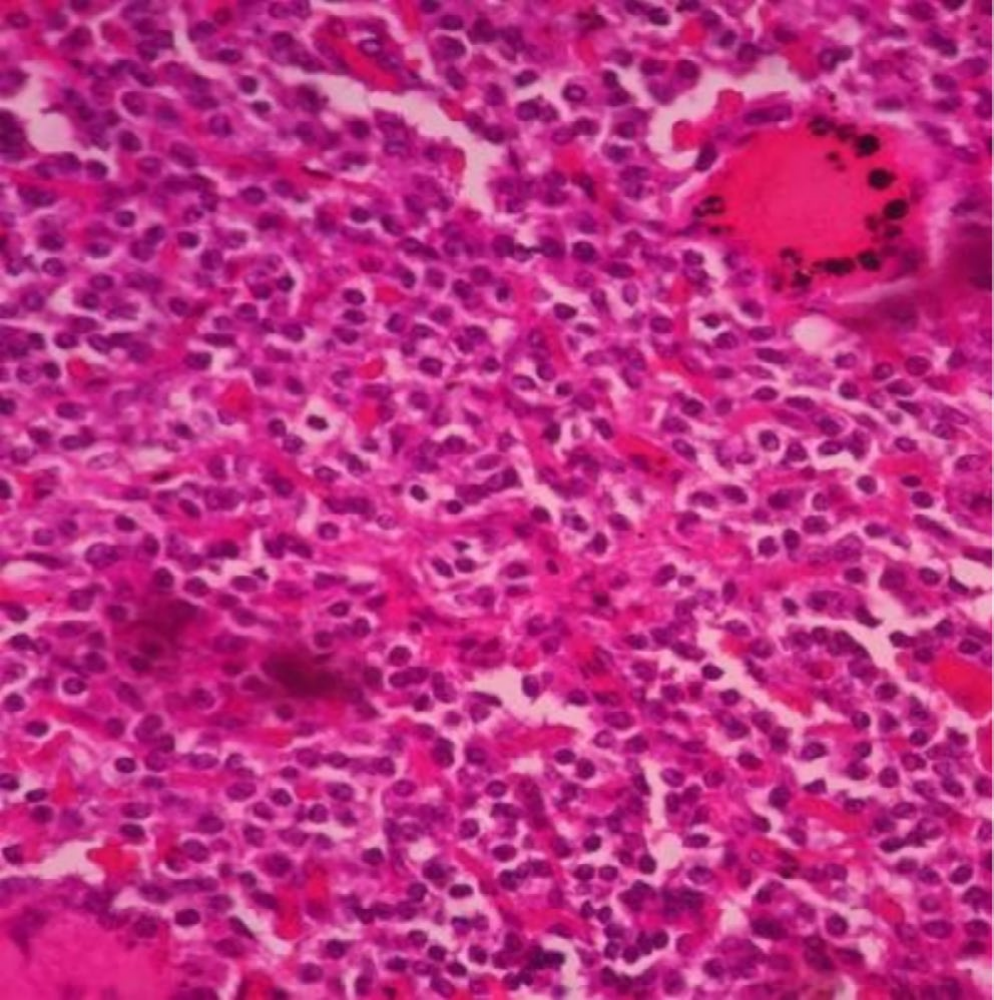
Histopathology. Hematoxilin and eosin 40×, tumor cells with oval to round nuclei and granular chromatin.

**FIGURE 4:**
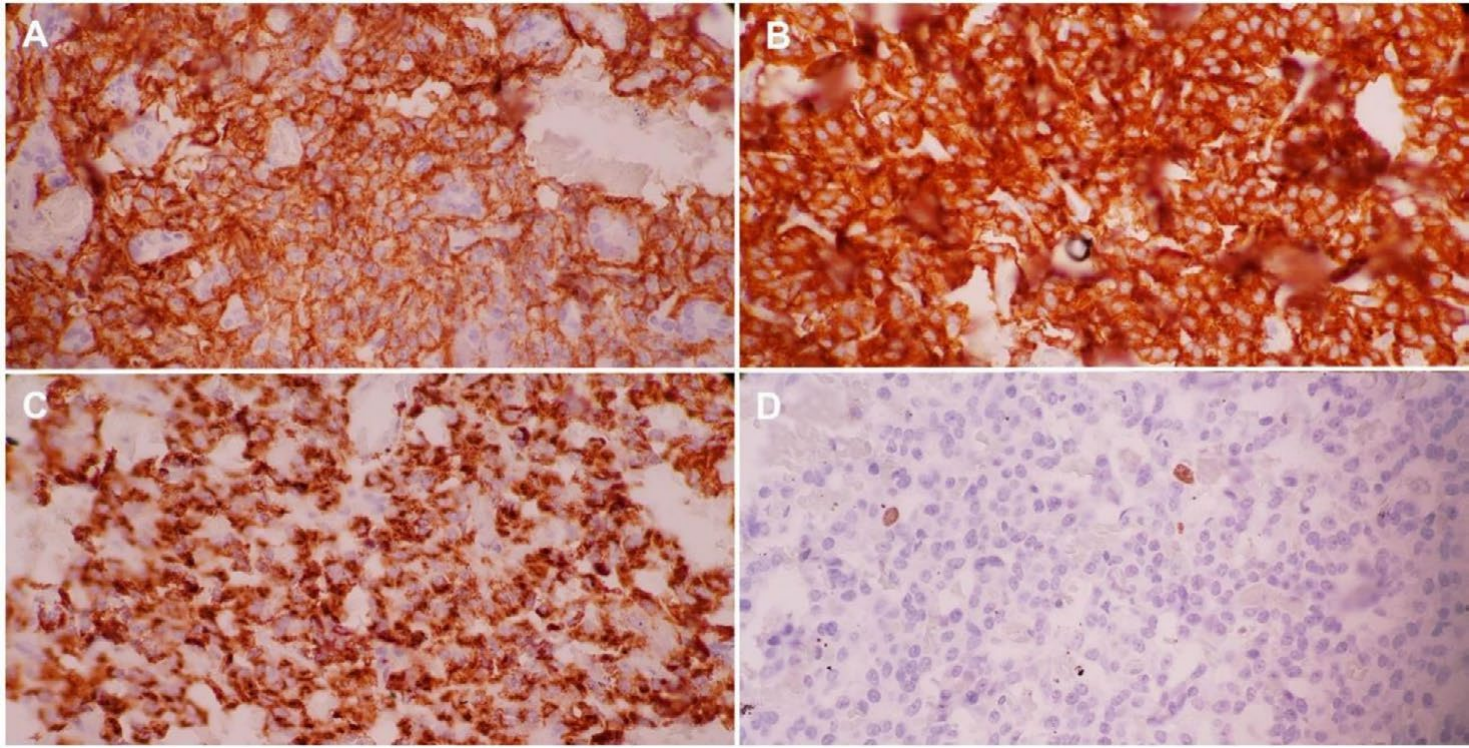
Immunohistochemistry. (A) Tumor cells exhibiting widespread cytoplasmic reactivity for chromogranin A. (B) Tumor cells displaying strong and diffuse cytoplasmic staining for synaptophysin. (C) Tumor cells with diffuse membranous expression of CD56. (D) Ki‐67 immunolabeling indicating a low proliferative index of approximately 2%.

A biopsy specimen obtained during surgery was submitted for histopathological examination, which revealed a well‐circumscribed nodular neoplasm. Microscopically, the lesion was composed of uniform, monotonous cells with round nuclei, moderate eosinophilic cytoplasm, and the characteristic “salt‐and‐pepper” chromatin pattern (Figure 3). Immunohistochemical staining was positive for synaptophysin and chromogranin A, confirming the diagnosis of a pancreatic neuroendocrine tumor (Figure 4).

## 4 Conclusion and Results (Outcome and Follow‐Up)

Following surgery, the patient experienced complete resolution of both hypoglycemic episodes and seizures. At the six‐month follow‐up, he remained asymptomatic, with marked cognitive improvement and no evidence of tumor recurrence on imaging.

Given the early age of presentation, the patient was referred for genetic counseling to rule out Multiple Endocrine Neoplasia type 1 (MEN‐1), although the tumor was solitary and no other clinical features were present at the time of this report.

## 5 Discussion

This case highlights the diagnostic challenge of insulinoma when neurological symptoms predominate. The 18‐month delay in diagnosis reflects the difficulty described in the literature, where the median time to diagnosis may span years due to the nonspecific nature of neuroglycopenic symptoms [6]. In our patient, this delay was primarily driven by the focal nature of the seizures, which strongly mimicked a structural neurological etiology (frontal lobe epilepsy) [8, 9]. Consequently, the initial clinical focus remained on seizure management, and the lack of early comprehensive endocrine testing suggests that the metabolic origin was overshadowed by the severity of the neurological presentation.

Although hypoglycemia typically causes diffuse neurological dysfunction, focal manifestations such as partial seizures can occur due to “selective neuronal vulnerability,” where brain regions with higher metabolic demand such as the cerebral cortex and hippocampus are more susceptible to glucose deprivation [10]. During hypoglycemia, ATP depletion leads to ionic pump failure and massive release of glutamate, causing excitotoxicity and creating an epileptogenic focus [10]. In this case, the right frontal lobe likely served as the focal point for seizure activity.

Neurologists must consider metabolic causes when seizures exhibit atypical features. Historical “red flags,” such as episodes occurring during fasting and improvement after glucose administration, should prompt evaluation for hypoglycemia [3].

The supervised 72‐h fasting test remains the gold standard for diagnosing endogenous hyperinsulinism [7]. According to the Endocrine Society criteria, diagnostic thresholds include glucose < 55 mg/dL with inappropriately elevated insulin (≥ 3.0 μU/mL) and C‐peptide (≥ 0.6 ng/mL). Elevated C‐peptide distinguishes endogenous from factitious hypoglycemia.

Accurate tumor localization is essential after biochemical confirmation. MRI was selected as the first‐line modality due to its superior soft‐tissue contrast resolution compared to computed tomography (CT) [11], and to avoid ionizing radiation given the patient's young age. Abdominal MRI has reported sensitivity of up to 85%, and insulinomas typically demonstrate marked arterial‐phase enhancement [12]. While other modalities such as Endoscopic Ultrasound (EUS) or 68Ga‐DOTATATE PET/CT are valuable for occult lesions, MRI served as an effective and non‐invasive initial approach. Endoscopic ultrasound (EUS) is another highly sensitive modality, particularly for lesions in the pancreatic head [13, 14].

Surgical resection is the treatment of choice for localized insulinomas, with cure rates exceeding 90% [15]. Distal pancreatectomy is safe and effective for tumors located in the pancreatic body or tail, and laparoscopic approaches offer advantages such as reduced blood loss and shorter hospital stays compared to open surgery [16].

The young age at presentation necessitates evaluation for MEN‐1, an autosomal dominant syndrome caused by mutations in the MEN1 gene [17]. Diagnosis has implications for lifelong surveillance of other associated tumors (parathyroid, pituitary) and for first‐degree relatives [18].

Oncologic prognosis for resected, well‐differentiated grade 1 neuroendocrine tumors is excellent, with cure rates above 95% [15]. The main long‐term concern is neurological sequelae from chronic neuroglycopenia. Fortunately, prospective studies show significant cognitive improvement after curative surgery, with many patients achieving full recovery [19].

Early recognition of insulinoma is crucial in patients presenting with recurrent focal seizures and neuroglycopenic symptoms, as misinterpretation as a primary neurological disorder may delay treatment and increase the risk of irreversible neurological damage. Considering hypoglycemia as part of the differential diagnosis allows timely intervention and improved outcomes.

## Author Contributions

**María Valerio López:** conceptualization, data curation, formal analysis, funding acquisition, investigation, methodology, project administration, resources, supervision, validation, visualization, writing – original draft. **Christopher Kaleb Romero Ríos:** conceptualization, data curation, formal analysis, funding acquisition, investigation, project administration, software, validation, visualization, writing – review and editing. **Mohammed Zahran:** conceptualization, data curation, formal analysis, investigation, methodology, project administration, supervision, writing – original draft. **Allan Mariano Bodan Campbell:** conceptualization, data curation, formal analysis, investigation, methodology, project administration. **Amilcar Alfaro:** conceptualization, data curation, formal analysis, investigation, methodology, writing – original draft. **Jorge Chamorro Flores:** conceptualization, data curation, formal analysis, funding acquisition, investigation, methodology, project administration, validation.

## Funding

The authors have nothing to report.

## Ethics Statement

Ethical approval was not required for this case report as per the regulations of the local ethics board, given the descriptive nature of the study and anonymization of patient data.

## Consent

Written informed consent was obtained from the patient for the publication of this case report and accompanying images.

## Conflicts of Interest

The authors declare no conflicts of interest.

## Data Availability

The data supporting the findings of this study are available from the corresponding author upon reasonable request. No datasets were generated or analyzed during the preparation of this case report.
